# Ileo-Ileal Knotting: A Rare Case of Bowel Strangulation

**DOI:** 10.7759/cureus.84060

**Published:** 2025-05-13

**Authors:** Valeria Leal Isla Flores, Alfredo S. Abarca Magallon, Yosira G López Alvarado, Cynthia L Nava Palomo, Carlos Iskyam Zaldo Arredondo

**Affiliations:** 1 General Surgery, ISSSTE (Instituto de Seguridad y Servicios Sociales de los Trabajadores del Estado) Clínica Hospital Constitución, Monterrey, MEX; 2 Coloproctology, ISSSTE (Instituto de Seguridad y Servicios Sociales de los Trabajadores del Estado) Regional Hospital Lic. Adolfo Lopez Mateos, Mexico City, MEX; 3 General Surgery, Hospital General De León, Leon, MEX

**Keywords:** acute abdomen, anastomosis, bowel obstruction, emergency exploratory laparotomy, ileo-ileal knotting, intestinal knot syndrome, resection, small bowel, surgery

## Abstract

Intestinal knotting syndrome is a rare cause of bowel obstruction, and ileo-ileal knotting is the least common type. Prompt surgical management is required to prevent rapid clinical deterioration. We present the case of a 50-year-old woman from Mexico arriving at the emergency room with intense abdominal pain, accompanied by nausea, vomiting, and mild abdominal distention. An exploratory laparotomy was performed, with the finding of a true ileo-ileal knot with ischemic bowel compromise. The etiology of ileo-ileal knotting is not well described, and it has not been linked to a specific age or sex. The surgical approach could vary between untying the knot if the affected loop is still viable or performing a resection en bloc with anastomosis if there are signs of necrosis or gangrene. Intestinal knotting is a diagnostic challenge usually confirmed at the time of the surgery. Surgeons must be aware of ileo-ileal knotting as a differential diagnosis for acute abdomen in order to operate on time, know when a resection is needed, and the right technique to perform in these cases.

## Introduction

Intestinal obstruction is usually caused by small bowel adhesions and represents a leading cause of emergency laparotomy [1,2]. First described by Riverius in the 16th century and later reported by Rokitansky in 1836 [3,4], ileo-ileal knotting is the twisting of a mobile bowel loop wrapping over another static bowel loop, forming a knot, causing obstruction and strangulation with a rapid clinical deterioration [5]. Other types of intestinal knot syndrome have been described, such as ileo-sigmoid (the most common), apendico-ileal, and ileocecal [6], but ileo-ileal knotting has the lowest reported incidence in the literature [2,7,8]. 

The etiology and predisposing factors for intestinal knot syndrome remain unclear [6], although some studies suggest that a mobile small bowel, long mesentery, decreased mesentery fat, a sudden bowel movement following the ingestion of a bulky or high fiber meal, usually after long periods of fasting, and even pregnancy, could be considered as contributing factors [9]. 

We present the case of a 50-year-old woman from Mexico arriving at the emergency room with acute abdomen, showing intense abdominal pain, accompanied by nausea, vomiting, and mild abdominal distention. The aim of this report is to contribute to the literature on ileo-ileal knotting and elucidate the appropriate surgical management depending on the transurgical findings to obtain a better outcome and lower the mortality rate of this pathology.

## Case presentation

A 50-year-old woman with irrelevant medical history aside from two pregnancies culminating in labor, denying any other medical and family pathological history, presented at the emergency department with a four-hour progression of generalized abdominal pain, with rising intensity, classified as 10/10 on the visual analogue scale, accompanied by nausea, gastrointestinal vomiting, malaise, with no bowel movements in the past 24 hours. Upon examination, she was tachycardic, tachypneic, and afebrile, with blood pressure within normal limits. The abdomen appeared slightly distended, with reduced peristaltic sounds, tympanic to percussion, muscle guarding, and rebound tenderness to palpation. Digital rectal examination was normal. Laboratory workup showed only mild leukocytosis with neutrophilia. The diagnosis of acute abdomen was made on clinical findings. She was started on IV antibiotics (ceftriaxone and metronidazole), analgesia, fluid resuscitation, and a nasogastric tube. 

**Figure 1:**
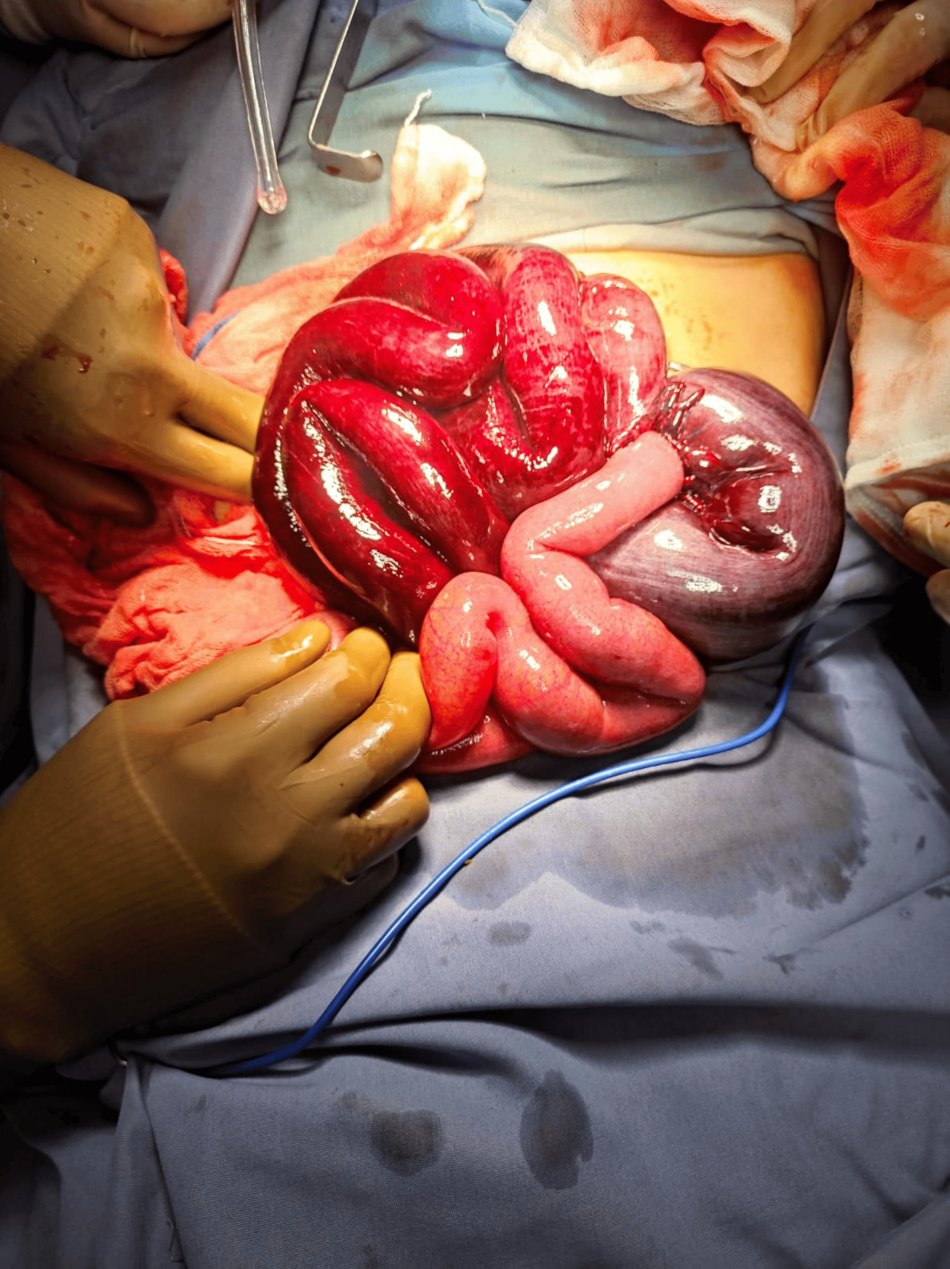
Exteriorization of the ileo-ileal knot showing segments of ischemia and necrosis.

**Figure 2:**
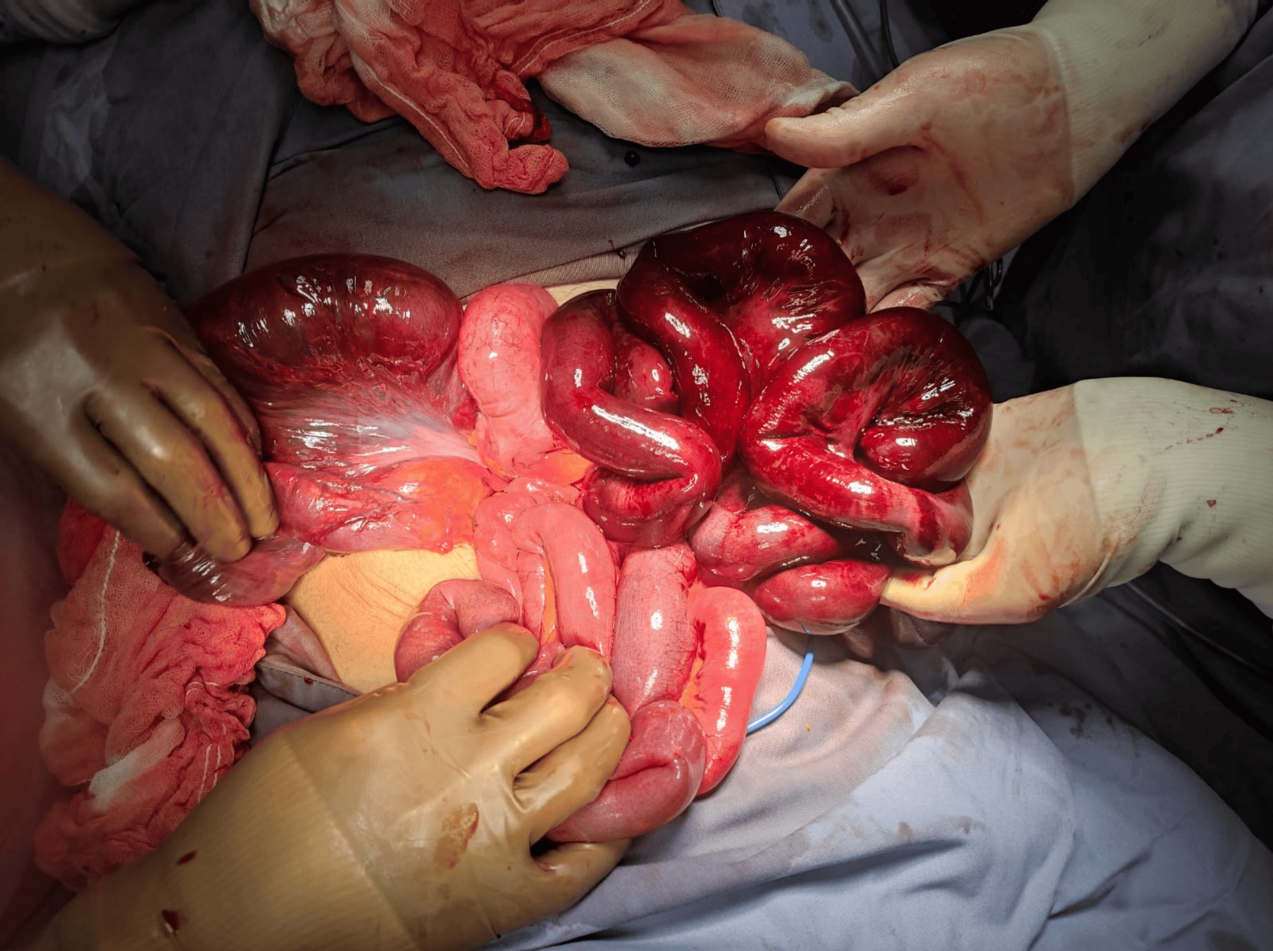
Ileal loop passing through another ileal segment with no ischemic repercussions.

An emergency laparotomy was performed within five hours of symptom onset. Intraoperative findings showed inflammatory free fluid and an ischemic bowel loop with necrotic segments (Figures 1, 2) of approximately 70 cm (27.56 inches) in length at 230 cm (90.55 inches) from the Treitz ligament and 300 cm (118.11 inches) from the ileocecal valve.

**Figure 3:**
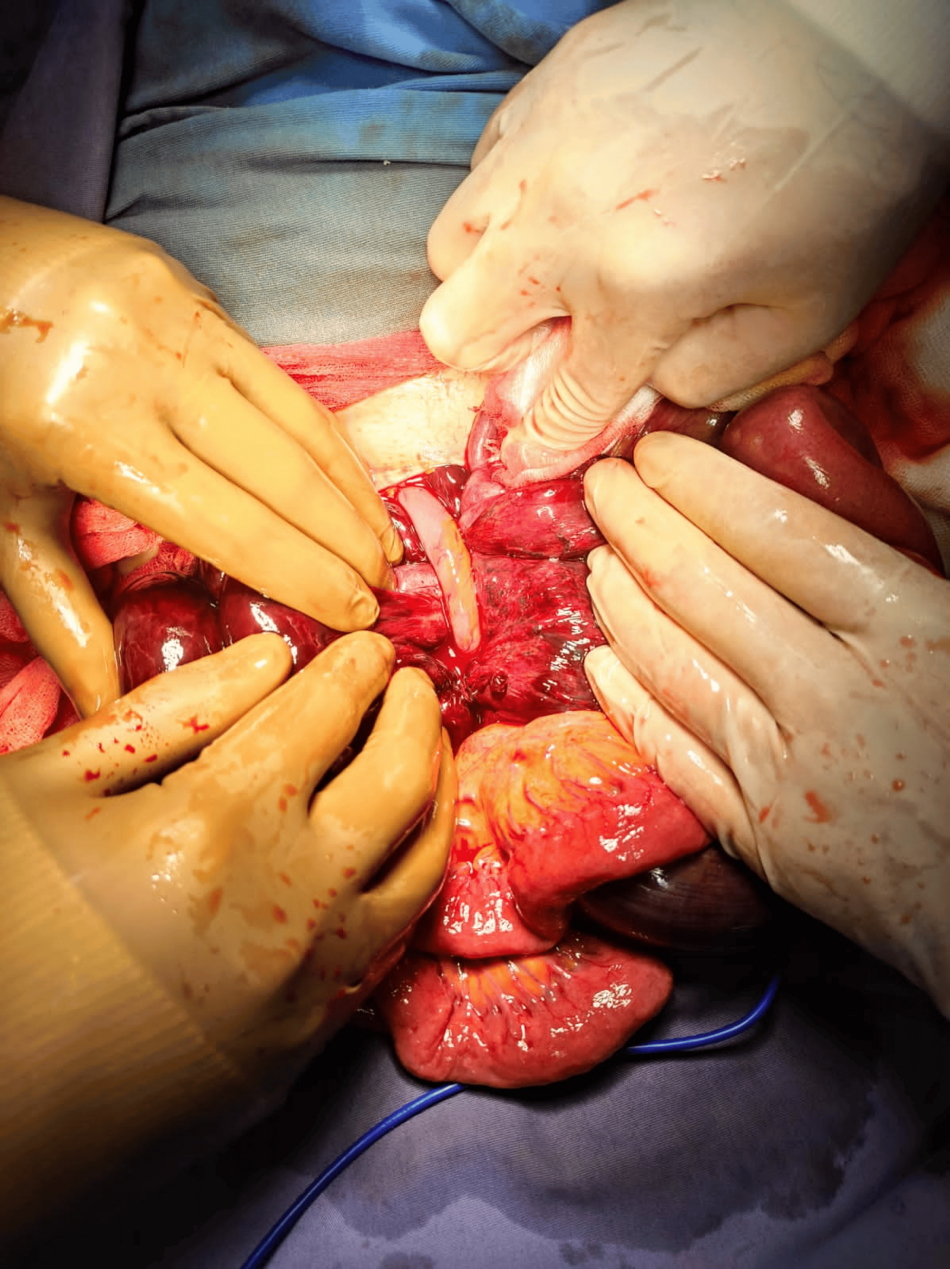
A true ileo-ileal knot, defining the fixed loop that caused the knot formation.

**Figure 4:**
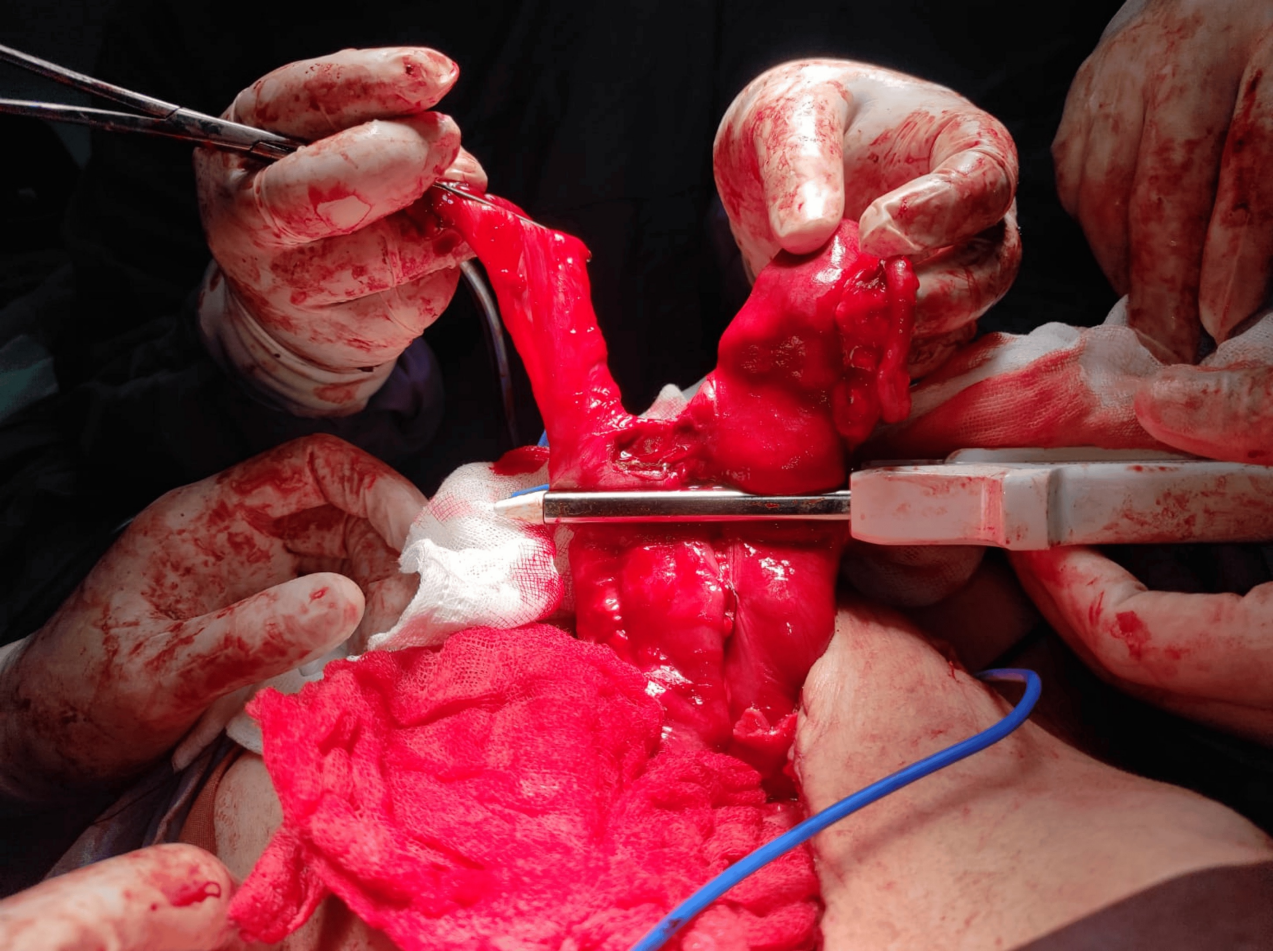
Mechanical ileo-ileal anastomosis.

It was evident how a fixed bowel loop by an adhesion encircled a portion of the ischemic bowel (Figure 3). Resection and mechanical latero-lateral, ileo-ileal anastomosis were performed (Figure 4) without untying the bowel knot because the affected intestine was not salvageable.

A thorough abdominal cavity lavage was performed, and a suction drain was inserted, directed to the pelvic cavity. The patient started oral feeding and ambulation 24 hours after the procedure, and her clinical course was favorable. She was discharged on the third postoperative day without the drain, and no complications were reported on outpatient follow-up.

## Discussion

Intestinal knot syndrome has been reported in patients as young as 11 months of age and as old as 80 years of age, unlinked to a specific sex [2]. It is more frequently observed in African and Asian populations, some identifying them as the “volvulus belt” countries [2,5,6,8,10-15]. Intestinal obstruction results in a rapid rise of intramural pressure, decreased mucosal perfusion because of the continued bowel peristalsis, lymphatic, venous, and arterial obstruction with progressive ischemia, subsequent necrosis, and a high risk of perforation, leading to peritonitis and sepsis [5,7,11,12]. All this could trigger effects like systemic inflammatory response syndrome (SIRS), metabolic acidemia, and electrolyte imbalances that contribute to a high mortality [7,14].

The ileo-ileal knot can be a true knot, meaning that a bowel loop passes through another intestinal segment, or it can be a pseudo knot, when the intestinal segments are intertwined without a segment crossing through another bowel loop [7,16]. It is hypothesized that increased peristalsis and a partial fixation of a bowel loop with an adhesion could lead to the knot formation [2,7,9,13,16]. Although the clinical presentation of ileo-ileal knotting is non-specific, displaying symptoms of bowel obstruction such as abdominal pain, nausea, vomiting and constipation/obstipation, a study reports the absence of significant abdominal distention, like the patient discussed in our case, as an observation that can help differentiate from a primary volvulus or other intestinal obstruction etiologies [9]. The importance of early diagnosis, or at least early surgical management in a clinical suspicion, falls on the rapid deterioration of the patient due to bowel necrosis, leading to a high mortality rate of 50% [5,11]. Initial management with IV fluid resuscitation, nasogastric decompression, pain control, and broad-spectrum antibiotics is also essential for a better outcome [2,5,9-12,15].

In our case, surgical management was based solely on clinical presentation, specifically signs of acute abdomen suggestive of intestinal pathology, with the definitive diagnosis made intraoperatively. The use of X-rays or ultrasound is not recommended due to their low reliability in making a diagnosis [7]; however, both could be used to differentiate whether the obstruction is from the small or large bowel [16]. CT scans have a sensitivity of 83% and a specificity of 93% and some findings reported in the literature include dilation of a bowel loop, air-fluid levels, bowel wall enhancement, “whirls sign” on the mesentery that can appear doubled as an “S” shape, intestinal pneumatosis and pericolic fluid [5-7,9,16].

As for surgical management, it is described that the first approach is to identify whether the bowel is viable or not. If the bowel is viable, it is advised to try to untie the knot and preserve as much bowel length as possible [6,8,16]. Some studies report that decompression by enterotomy of the affected bowel loop makes it easier to untie a tight knot [2,16]. If the bowel is already ischemic or necrotic, it is better to make an en bloc resection without untying the knot, ligating the mesenteric vessels first, to prevent perforation with spillage of contents and the dissemination of proinflammatory cytokines (interleukin 6 (IL-6), tumor necrosis factor alpha (TNF-α)) to the circulation [2,6-9,11,13]. There is also an important loss of the intestinal mucosa within 45 minutes of ischemia, favoring the exposure of pathogens to the bowel wall, the activation of the complement cascade, the overexpression of endothelial adhesion molecules promoting neutrophil sequestration and propagating the epithelial damage [17]. 

## Conclusions

Ileo-ileal knotting is a rare but severe form of intestinal obstruction that requires prompt surgical intervention to prevent irreversible bowel ischemia and high mortality. Early diagnosis relies heavily on clinical suspicion and intraoperative assessment, as imaging modalities have limited diagnostic reliability in acute settings. Surgical management should prioritize the assessment of bowel viability, with resection of necrotic segments and avoidance of unnecessary manipulation of the knot to reduce further complications. Timely intervention and appropriate surgical strategies are essential to improve patient outcomes and reduce the risk of life-threatening sequelae associated with this uncommon but critical condition.

## References

[1] Ten Broek RP, Krielen P, Di Saverio S (2018). Bologna guidelines for diagnosis and management of adhesive small bowel obstruction (ASBO): 2017 update of the evidence-based guidelines from the world society of emergency surgery ASBO working group. World J Emerg Surg.

[2] Molla YD, Mequanint MB, Bisrat SH, Workneh GA, Alemu HT (2024). Ileo-ileal knot causing acute gangrenous small bowel obstruction: a case report. J Med Case Rep.

[3] Uday SK, Venkata PK, Bhargav PR, Kumar S (2012). Ileo-ileal knot causing small bowel gangrene: an unusual presentation. Int J Case Rep Image.

[4] Rokitansky C (1836). About internal intestinal strictures [Article in German]. Osterr Med Jahrbücher.

[5] Dallakoti N, Pant VP, Pokhrel N, Bhatta P, Rana VK, Sah RP (2024). Ileocecal knotting as a rare cause of small bowel obstruction: a case report. Ann Med Surg (Lond).

[6] Ewnte B, Girma E (2022). Ileo-sigmoid knotting in a female Ethiopian patient, a case report. Int J Surg Case Rep.

[7] Harini JJ, Satish JK, Rajeswari PA, Prabhu GG (2024). Ileo-ileal knotting: a ticking bomb. Indian Pediatr.

[8] Mohammed M, Wondimu B, Abera E (2023). A rare case report of viable ileo-ileal knotting of acute abdomen in adults. Int J Surg Case Rep.

[9] Idowu NA, Ismaeel WO, Adeleke AA, Faleye JA, Adeleye-Idowu SA, Ademoye KA (2024). Appendico-Ileal knotting: a rare cause of strangulated small bowel obstruction. Ethiop J Health Sci.

[10] Mushtaq S, Tan Su Yin A, Sze Kiat S, Azim Nik Abdullah N (2020). Intestinal knotting: a case report and brief literature review. Med J Malaysia.

[11] Rahimi-Movaghar E, Tahouri T (2022). Ileo-sigmoid knotting- an unusual cause of intestinal obstruction: a case report. Int J Surg Case Rep.

[12] Tena Shale W, James Oriho L (2023). A rare case of Ileo-Ileal knotting: a case report. Cureus.

[13] Subedi SS, Gupta RK, Neupane D, Agrawal S, Khanal B, Jaiswal LS (2022). Ileosigmoid knotting: a case report of unique cause of acute abdomen in a Nepalese patient. Int J Surg Case Rep.

[14] Kabuye U, Damulira J, Okuku MD (2024). Appendico-ileal knot: a rare form of small bowel obstruction: a case report. Int J Surg Case Rep.

[15] Abule T, Chebo T, Billoro BB (2022). Appendico-ileal knotting causing small bowel obstruction: a case report. Clin Case Rep.

[16] Isamu S, Touge R, Kojima M, Kurihara S, Tani C (2023). A case of strangulated intestinal obstruction due to ileo-ileal true knot: peculiar computed tomography findings. J Ped Surg Case Rep.

[17] Grootjans J, Lenaerts K, Derikx JP (2010). Human intestinal ischemia-reperfusion-induced inflammation characterized: experiences from a new translational model. Am J Pathol.

